# High arrhythmic risk in antero-septal acute myocardial ischemia is explained by increased transmural reentry occurrence

**DOI:** 10.1038/s41598-019-53221-2

**Published:** 2019-11-14

**Authors:** Hector Martinez-Navarro, Ana Mincholé, Alfonso Bueno-Orovio, Blanca Rodriguez

**Affiliations:** Department of Computer Science, British Heart Foundation Centre of Research Excellence, University of Oxford, Parks Rd., OX13QD Oxford, UK

**Keywords:** Acute coronary syndromes, Translational research

## Abstract

Acute myocardial ischemia is a precursor of sudden arrhythmic death. Variability in its manifestation hampers understanding of arrhythmia mechanisms and challenges risk stratification. Our aim is to unravel the mechanisms underlying how size, transmural extent and location of ischemia determine arrhythmia vulnerability and ECG alterations. High performance computing simulations using a human torso/biventricular biophysically-detailed model were conducted to quantify the impact of varying ischemic region properties, including location (LAD/LCX occlusion), transmural/subendocardial ischemia, size, and normal/slow myocardial propagation. ECG biomarkers and vulnerability window for reentry were computed in over 400 simulations for 18 cases evaluated. Two distinct mechanisms explained larger vulnerability to reentry in transmural versus subendocardial ischemia. Macro-reentry around the ischemic region was the primary mechanism increasing arrhythmic risk in transmural versus subendocardial ischemia, for both LAD and LCX occlusion. Transmural micro-reentry at the ischemic border zone explained arrhythmic vulnerability in subendocardial ischemia, especially in LAD occlusion, as reentries were favoured by the ischemic region intersecting the septo-apical region. ST elevation reflected ischemic extent in transmural ischemia for LCX and LAD occlusion but not in subendocardial ischemia (associated with mild ST depression). The technology and results presented can inform safety and efficacy evaluation of anti-arrhythmic therapy in acute myocardial ischemia.

## Introduction

Acute myocardial ischemia is still one of the leading causes of sudden cardiac death worldwide^1^. It arises from a mismatch between supply and consumption of oxygen and nutrients, and poor waste removal, often due to narrowing of a coronary artery. One of the clinical challenges in the management of patients suffering from acute myocardial ischemia is its highly variable manifestation, due to differences in location, extent and severity of affected myocardium. Some patients exhibit marked ECG abnormalities, and specifically ST elevation, whereas others suffer almost unnoticeable changes on their ECG^2^. It is also unclear how ischemia-induced ECG abnormalities can be used effectively for arrhythmia risk stratification.

The first 10–15 minutes, or phase 1 A, of acute myocardial ischemia are particularly pro-arrhythmic^3^ due to increased heterogeneity of repolarisation and conduction around the ischemic region in the human ventricles^4^. These electrophysiological heterogeneities establish the pro-arrhythmic substrate for reentrant waves^5^, potentially breaking into ventricular fibrillation^6,7^. Computer simulations using human ventricular models have been able to reproduce existing knowledge on acute ischemia-induced electrophysiological alterations and the establishment of figure-of-eight reentrant dynamics around ischemic regions^5^.

This study aims to investigate how the size, transmural extent and location of the ischemic region modulate pro-arrhythmic mechanisms of reentrant dynamics in the human ventricles. We hypothesize that macro-reentrant patterns (such as figure-of-eight) are more likely to occur around large and fully transmural ischemic regions, whereas subendocardial ischemia sustains transmural reentrant patterns, especially in the anatomically-complex septal region following LAD occlusion. This research question has not been addressed so far^8–11^, due to severe methodological challenges. Firstly, experiments in human whole-ventricles are challenging due to ethical and practical limitations, and the spatiotemporal dynamics of acute regional ischemia continuously change the substrate. Secondly, computationally, the investigations require multiscale anatomically-based human torso/ventricular models, which are very costly and require high performance computing. In this study, we exploit high resolution datasets obtained from high performance computing simulations using a human torso/biventricular electrophysiology model, constructed and evaluated using extensive experimental and clinical data from ionic dynamics to the ECG. Electrophysiological changes in phase 1A ischemia are different from those observed in other stages post-occlusion, such as phase 1B ischemia or infarction, which have been the focus of previous investigations^12,13^.

## Methods

### Human torso/biventricular electrophysiological model in acute ischemia

**Figure 1:**
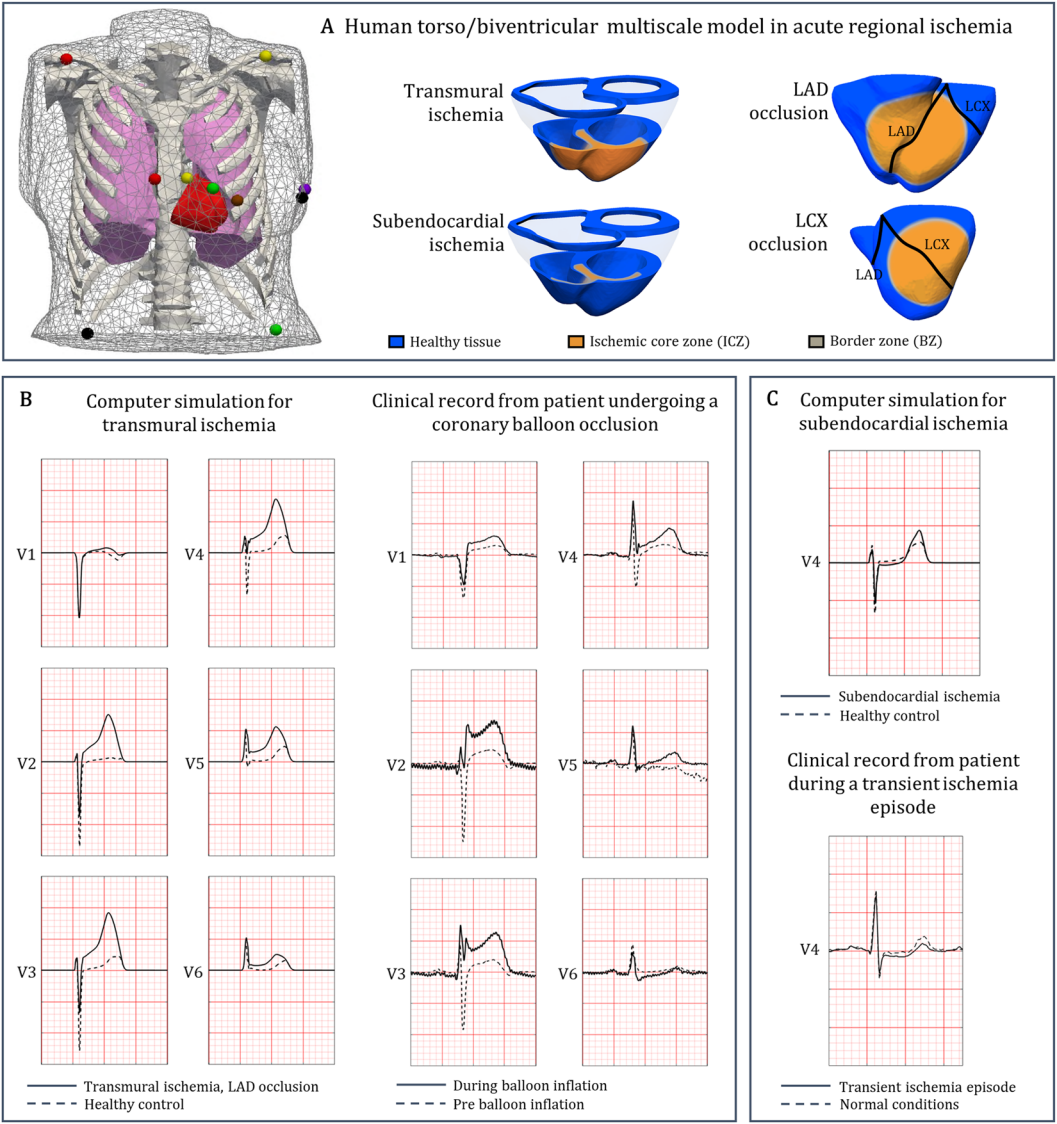
Human torso/biventricular electrophysiology model in acute regional ischemia for ECG and arrhythmia simulations. (**A**) Human torso/biventricular electrophysiology model in acute regional ischemia, with the 12-lead ECG electrode locations (with coloured spheres representing) using standard European colour-coding (left, reproduced from^14^ under open access license); schematic representation of LCX/LAD occlusion and transmural/subendocardial ischemia (right). (**B**) Comparison between simulated ECG signal in the precordial leads in transmural ischemia (left column, solid line) and clinical recording from a STAFF III database patient undergoing a LAD coronary balloon occlusion (right column, solid line). (**C**) Comparison between simulated ECG signal in the precordial leads in subendocardial ischemia (top, solid line) and clinical ECG from the Long-Term ST Database during transient ischemia episode (bottom, solid line). Dashed lines for ECG in control/pre-ischemia.

A human biventricular model embedded in a torso was used to simulate electrophysiological activity from ionic dynamics to body surface potentials (Fig. 1A, left)^14^. Electrophysiological alterations in the ischemic region were modelled as in previous studies^15,16^, including ischemic core zone (ICZ), lateral border zone (BZ) and endocardial BZ. Membrane dynamics were represented by the modified O’Hara-Rudy model^17,18^, as described in Supplementary Materials EM.1.

Electrophysiological effects of acute ischemia, caused by hyperkalemia, hypoxia and acidosis were introduced to reproduce the heterogeneous changes in refractoriness and conduction velocity in human ischemic tissue^17^. In total, 18 scenarios were simulated, including 16 cases of acute regional ischemia corresponding to combinations of two different sizes, two transmural extents (subendocardial and transmural ischemia), and two locations (representing LCX and LAD occlusion, respectively) (Fig. 1A and Supplementary Materials EM.2). In addition, as acute ischemia often occurs in patients with a diseased cardiac substrate, we investigated the impact of slow electrical propagation (by 25%) throughout the ventricles for all combinations. Further details on the construction and evaluation of the computational model are provided in Supplementary Materials EM.1 and EM.2.

### ECG simulations and comparison to clinical recordings

The 12-lead ECG was calculated at standard clinical electrode positions on the torso (Fig. 1A, left). ST deviation and QRS slopes were computed, as important indicators of ischemia-induced electrophysiological abnormalities^19,20^. The change in QRS downslopes in comparison to control (ΔQRS_DS_) was quantified, with positive values indicating flatter slopes in ischemia. Simulated ECGs and measured biomarkers were compared with clinical recordings from the Physionet repository (https://physionet.org/), and specifically the STAFF database, collected with Institutional Review Board approval^21^. Additional details are provided in Supplementary Material EM.3.

### Stimulation protocols

Sinus rhythm was simulated using a realistic activation sequence by applying endocardial stimulation^14^ for 3 beats (S1) with a cycle length of 600 ms. To evaluate reentry vulnerability, ectopic stimulation (S2) was applied transmurally at the BZ. This was based on the experimental evidence from^22^ reporting that the earliest activity of premature beats in acute myocardial ischemia was found to occur in the normal myocardium adjacent to the ischemic region, and that no important time differences were found between endo- and epicardium. S2 was applied at varying coupling intervals (CI, i.e. time difference between the last S1 and S2) and for each CI, simulated electrical activity was analysed to identify reentry occurrence. The vulnerability window (VW) was quantified as the range of CIs for which S2 resulted in at least two reentrant cycles. As the relative location of the ectopic stimulation in the ischemic BZ with respect to ventricular anatomy and ischemic region may influence the VW, we considered six S2 locations equally spaced around the BZ. Simulations were conducted using the numerical solver CHASTE^23^. By combining multiple scenarios, CIs and ectopic locations, we conducted a computationally-expensive study of more than 400 simulations of 3–11 hours on 720 CPUs.

### Data statement

The numerical solver CHASTE is freely available as Open Source at [http://www.cs.ox.ac.uk/chaste/download.html]. Meshes, models, and scripts to replicate simulation outputs can be found at [10.5287/bodleian:9RxJPo9po].

## Results

### Credibility of human torso-ventricular model for ECG and reentry vulnerability investigations in control and acute ischemia

Simulation results with the human torso-biventricular model in acute ischemia were first evaluated through comparison with experimental and clinical recordings. Firstly, as demonstrated in our previous study^17^ and in Supplementary Tables ST1 and ST2, the electrophysiological consequences of acute myocardial ischemia at ionic, cellular and tissue level are reproduced in the simulations with the human ventricular tissue model, in agreement with experimental recordings^3,4,17,24,25^. Specifically, in the simulations, ischemic tissue exhibits human ventricular action potential duration shortening, elevation of resting potential, prolonged post-repolarization refractoriness and decreased conduction velocity (see Supplementary Tables ST1 and ST2) as shown in previous experimental, clinical and computational recordings^3,4,17,24–28^. These are the key electrophysiological properties relevant for evaluation of reentrant dynamics and ECG changes in acute ischemia.

Furthermore, as described in our previous study^14^, the endocardial activation model imposed to simulate sinus rhythm yields activation sequence and QRS complex in the 12-lead ECG, consistent with experimental and clinical recordings^29–31^. This is further illustrated in Supplementary Fig. SF2, which shows the agreement of simulated ECG in healthy conditions compared to a clinical recording from a healthy volunteer from the PTB database^32^, both in terms of QRS complex and T wave morphology.

Introduction of acute regional ischemia in the human torso/biventricular model causes QRS alterations and ST deviations in agreement with clinical recordings for both transmural and subendocardial ischemia (Fig. 1B,C, respectively). ST elevation values obtained in simulated transmural ischemia (274 to 319 µV, leads V2, V3 and V4; Fig. 1B, left) are within the range obtained clinically during coronary balloon LAD occlusion (200 to 500 µV in leads V2, V3 and V4, Fig. 1B, right)^21^. Figure 1C confirms agreement between simulated and clinical ECGs under subendocardial ischemia, displaying mild ST depression (simulations: 28 µV in lead V2; −6.3 µV in lead V3; −25 µV in lead V4) in range with maximal depression of up to −85 µV in clinical transient ischemic ECGs^33^. Thus, the consistency of simulation results with experimental and clinical recordings for ionic, cellular, tissue, whole-ventricular and ECG properties yields credibility to the findings presented below.

### Impact of location, transmural extent and size of acute regional ischemia on ECG biomarkers

**Figure 2:**
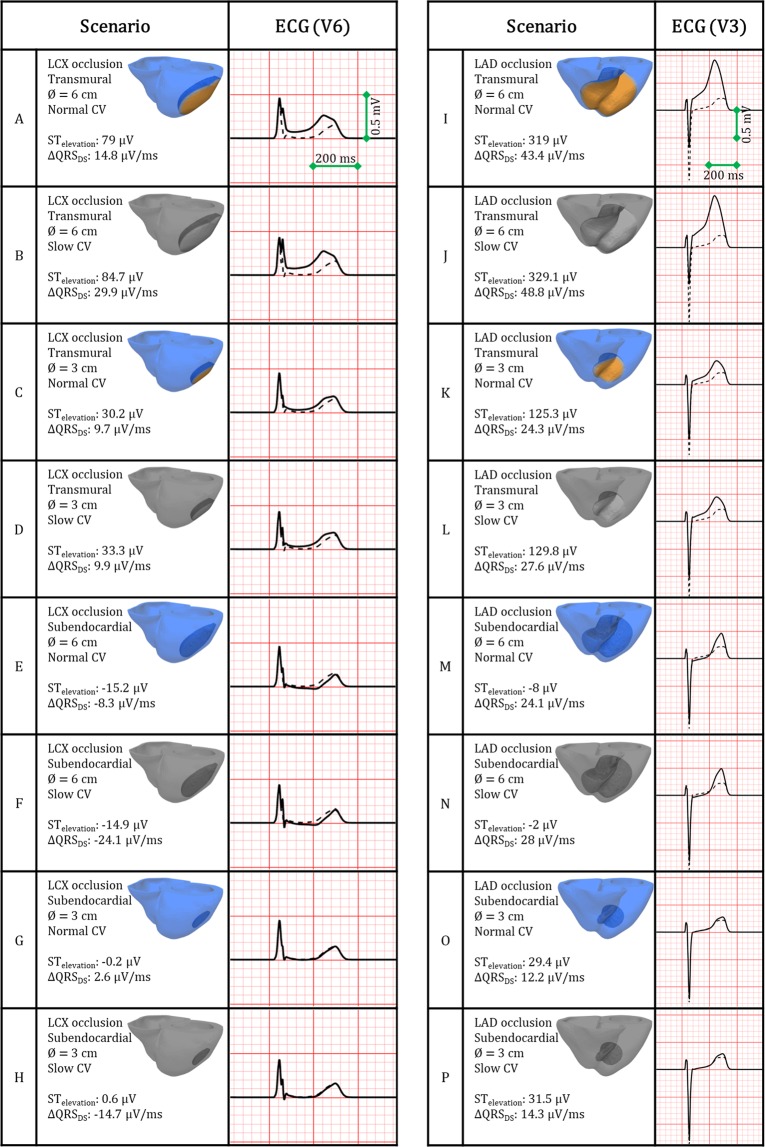
ECG alterations caused by acutely-ischemic regions, for LCX versus LAD occlusion (**A–H** and **I–P**, respectively), transmural (**A–D** and **I–L**) versus subendocardial (**E–H** and **M–P**) ischemia, small versus large ischemic region diameters (∅ = 3 and 6 cm, respectively), and normal versus reduced propagation in remote myocardium (full colour and grayscale, respectively). Dashed line indicates the ECG under control/pre-ischemia. Leads V6 for LCX and V3 for LAD occlusions are shown with ST deviation and change in maximum QRS downslope.

Figure 2 shows simulated ECGs for the 16 ischemic scenarios for leads V6 (LCX occlusion, cases A-H) and V3 (LAD occlusion, cases I-P), chosen due to the spatial proximity of those leads to the lateral and anterior myocardial walls, respectively. Values of ST deviation and variation in maximum QRS downslope (ΔQRS_DS_) are provided. Overall, ECG alterations were more severe for LAD (Fig. 2, right) than for LCX occlusion (Fig. 2, left). Transmural ischemia caused marked ST elevation (Fig. 2A–D,I–L), whereas subendocardial ischemia primarily resulted in mild ST depression (Fig. 2E–H,M–P). Slow myocardial propagation (Fig. 2, grey shade scenarios) mildly prolonged the QRS interval with negligible effects on the ST segment.

**Figure 3:**
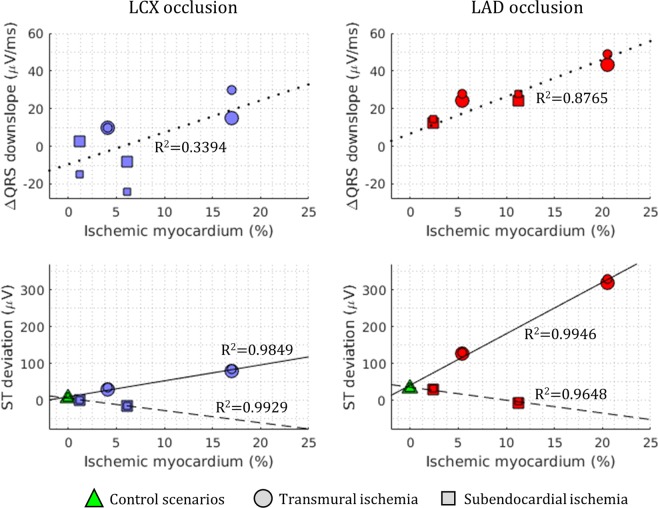
Ischemia-induced alterations in ECG biomarkers versus percentage of ischemic myocardium (top: change in maximum QRS downslope; bottom: ST segment deviation), for LCX occlusion (left column, blue scatter points) and LAD occlusion (right column, red scatter points). Coefficients of determination (R^2^) are annotated for each biomarker. Smaller symbols correspond to slow propagation conditions.

Figure 3 provides further quantification of ΔQRS_DS_ and ST deviation as a function of percentage of ischemic myocardium (top and bottom, respectively), for transmural (circles) and subendocardial (squares) ischemia. ΔQRS_DS_ positively correlates with the volume of ischemic myocardium for LAD occlusion (Fig. 3, top right panel), but not for LCX occlusion (Fig. 3, top left panel). For both LAD and LCX occlusion, ST elevation positively correlates with the size of the transmural ischemic region (circles, solid lines). However, subendocardial ischemia (squares) results in low or negligible ST depression in all cases regardless of location or size (dashed lines).

### Differences in wavelength for reentry explain the effect of ischemic region size and myocardial conduction velocity on arrhythmic risk

**Figure 4:**
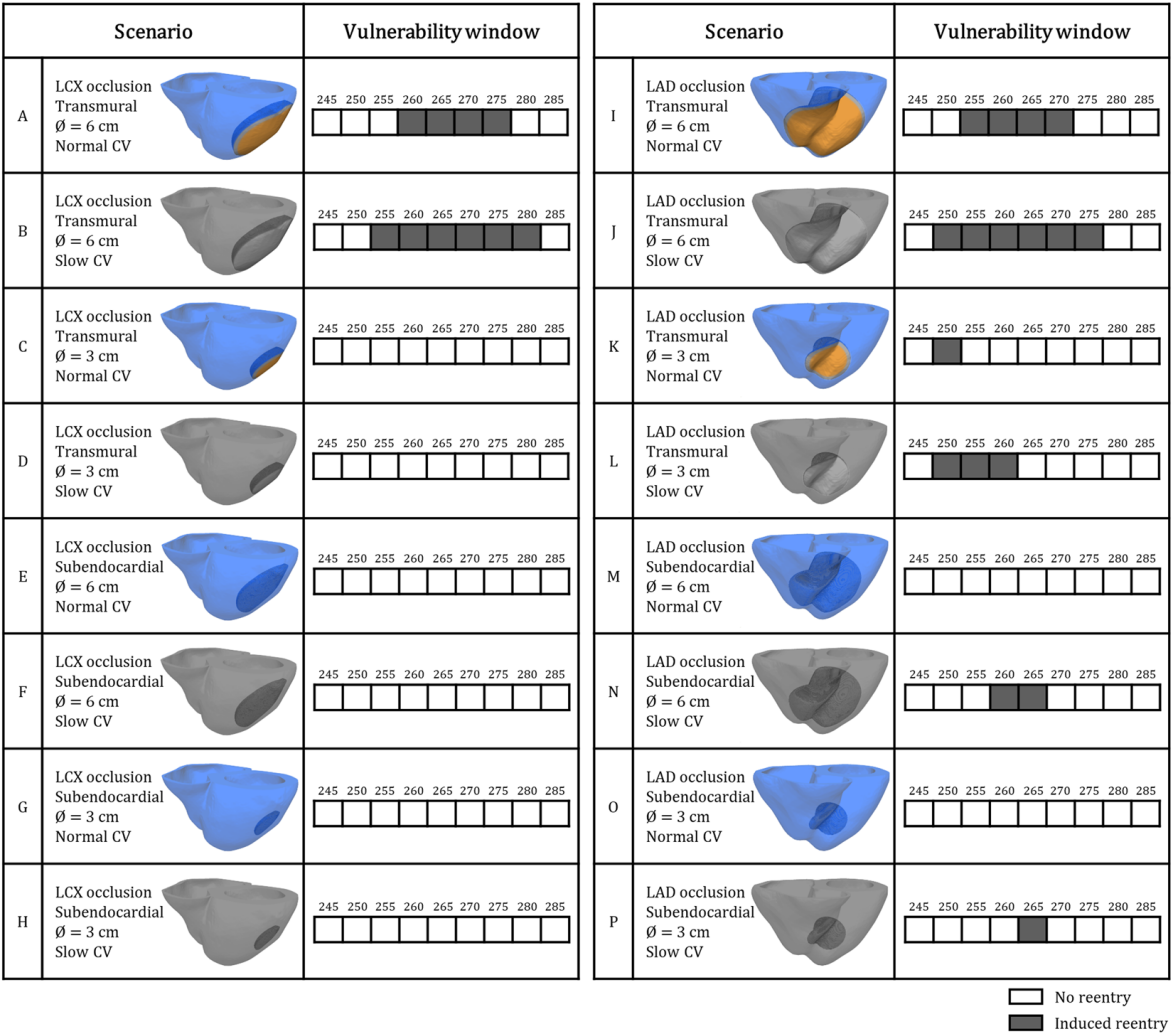
Vulnerability windows for reentry (dark grey boxes) in acute regional ischemia for LCX versus LAD occlusion (**A–H** and **I–P**, respectively), transmural (**A–D** and **I–L**) versus subendocardial (**E–H** and **M–P**) ischemia, small versus large ischemic region diameters, and normal versus reduced propagation in remote myocardium (full colour and grayscale, respectively). Ectopic stimulus applied at CI = 245 to 285 ms in ischemic border zone in LV mid-cavity (S2.b in Fig. 6).

Figure 4 displays the results of the VWs obtained for the 16 different ischemia scenarios with premature stimulus S2 in the ischemic BZ in the LV mid-cavity for both LAD and LCX occlusion. No reentry was induced without ischemia with the same protocol. Consistent with the theory on reentrant circuits, slow myocardial propagation (as imposed in the grey-coloured cases in Figs 2 and 4) facilitates reentry by reducing the wavelength of cardiac impulse (i.e. conduction velocity multiplied by refractory period). This is demonstrated in Fig. 4 by the wider VWs reported in each grey-coloured scenario with slow conduction velocity versus their correspondent case with normal conduction velocity in the remote myocardium. Additionally, larger ICZ were always more arrhythmogenic than smaller ones (Fig. 4).

**Figure 5:**
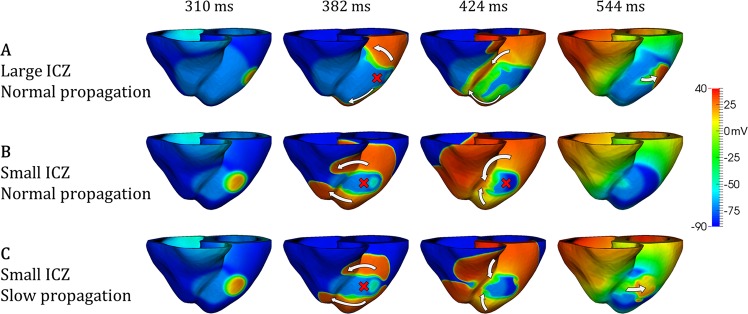
Effects of slow myocardial propagation and ischemic region size on macro-reentry formation. (**A**) Figure-of-eight macro-reentry pattern induced around large transmural ischemic region in LAD occlusion. (**B**) No reentry for small transmural ischemic region in LAD occlusion. (**C**) Similar case as in B, but macro-reentry is enabled due to slow myocardial propagation. CI = 260 ms in all cases.

The mechanisms are illustrated in Fig. 5, for large versus small ischemic regions (Fig. 5A,B, respectively), and normal versus slow myocardial propagation for small ischemic regions (Fig. 5B,C, respectively). In agreement with previous studies^5,15^, propagation following the ectopic stimulus is blocked in the still refractory ischemic region (red cross, 382 ms), but circles around it towards the RV through base and apex (white arrows, 382 ms). The larger pathway through the large ischemic region (Fig. 5A) allows enough time, firstly, for ischemic tissue to recover and to allow retrograde propagation through the large ischemic region (Fig. 5A, 424 ms), and secondly, for the normal tissue to recover once propagation has traversed the ischemic region, ensuring the continuation of the reentry (Fig. 5A, 544 ms).

In contrast, the small ischemic region (Fig. 5B) does not allow enough time for the recovery of ischemic tissue, preventing retrograde propagation and leading to bidirectional conduction block (Fig. 5B, 424 ms), and no reentry established (Fig. 5B, 544 ms). Reducing conduction velocity promotes the formation of reentrant circuits by delaying propagation around the ischemic region (Fig. 5C, 382 ms), allowing the recovery of the ischemic tissue and ensuring retrograde propagation and reentry (Fig. 5C, 424, 544 ms), even with small ischemic region.

### Arrhythmia vulnerability in subendorcardial LAD occlusion is explained by septo-apical transmural microreentrant pathways

**Figure 6:**
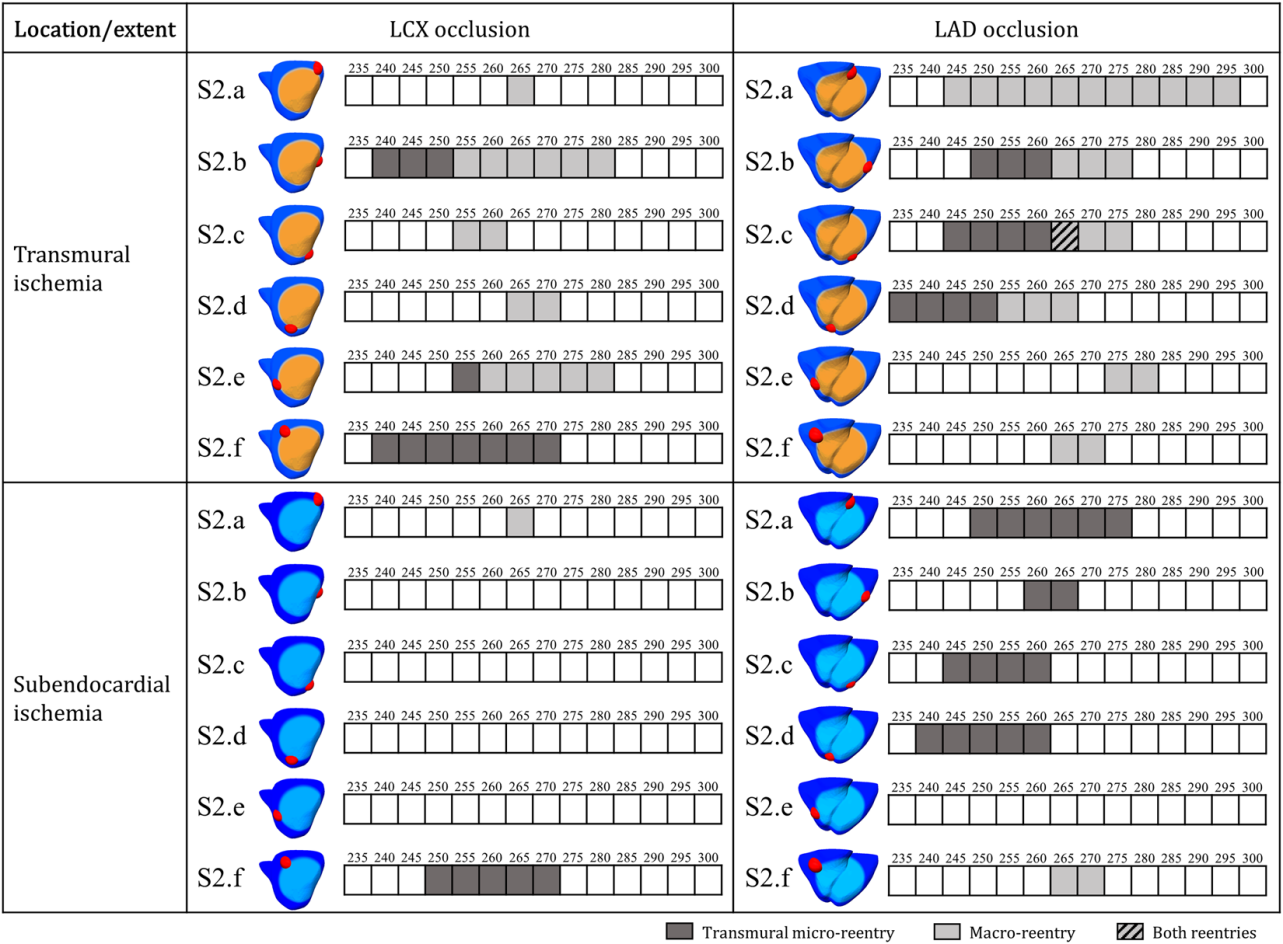
Vulnerability windows for reentry for the most pro-arrhythmic conditions, in LAD versus LCX occlusion and transmural versus subendocardial ischemic region for 6 equally-spaced premature stimulus locations around the ischemic region. Macro-reentry (light grey boxes), transmural micro-reentry (dark grey boxes) and no reentry (white boxes) are indicated for CI = 235 to 300 ms.

We then investigated the mechanisms explaining how the effect of location and transmural extent of the ischemic region may modulate arrhythmic risk. We hypothesized that the intersection of the ischemic border zone with the septum, as in LAD occlusion, promotes the establishment of transmural micro-reentrant pathways. In the investigation of differences between LAD and LCX occlusion, the location of the ectopic stimulus with respect to the LV anatomy is likely to modulate arrhythmic risk. We therefore computed the VWs for six different S2 locations around the BZ, for large transmural and subendocardial ischemic areas in LAD versus LCX occlusion (Fig. 6). Overall, more reentries are induced in LAD versus LCX occlusion (54 versus 33 reentries for CIs = 225–300 ms), considering both transmural and subendocardial ischemia, for the conditions tested. Furthermore, more reentries are established in transmural versus subendocardial ischemia: 27 versus 6 reentries, respectively, in LCX occlusion, and 35 versus 19 reentries, respectively, in LAD occlusion (Fig. 6).

Macro-reentry around the ischemic region, as described in Fig. 5 and in previous studies^5,15^, was established in transmural ischemia, for both LCX and LAD occlusions and most S2 locations (Fig. 6, light grey boxes).

**Figure 7:**
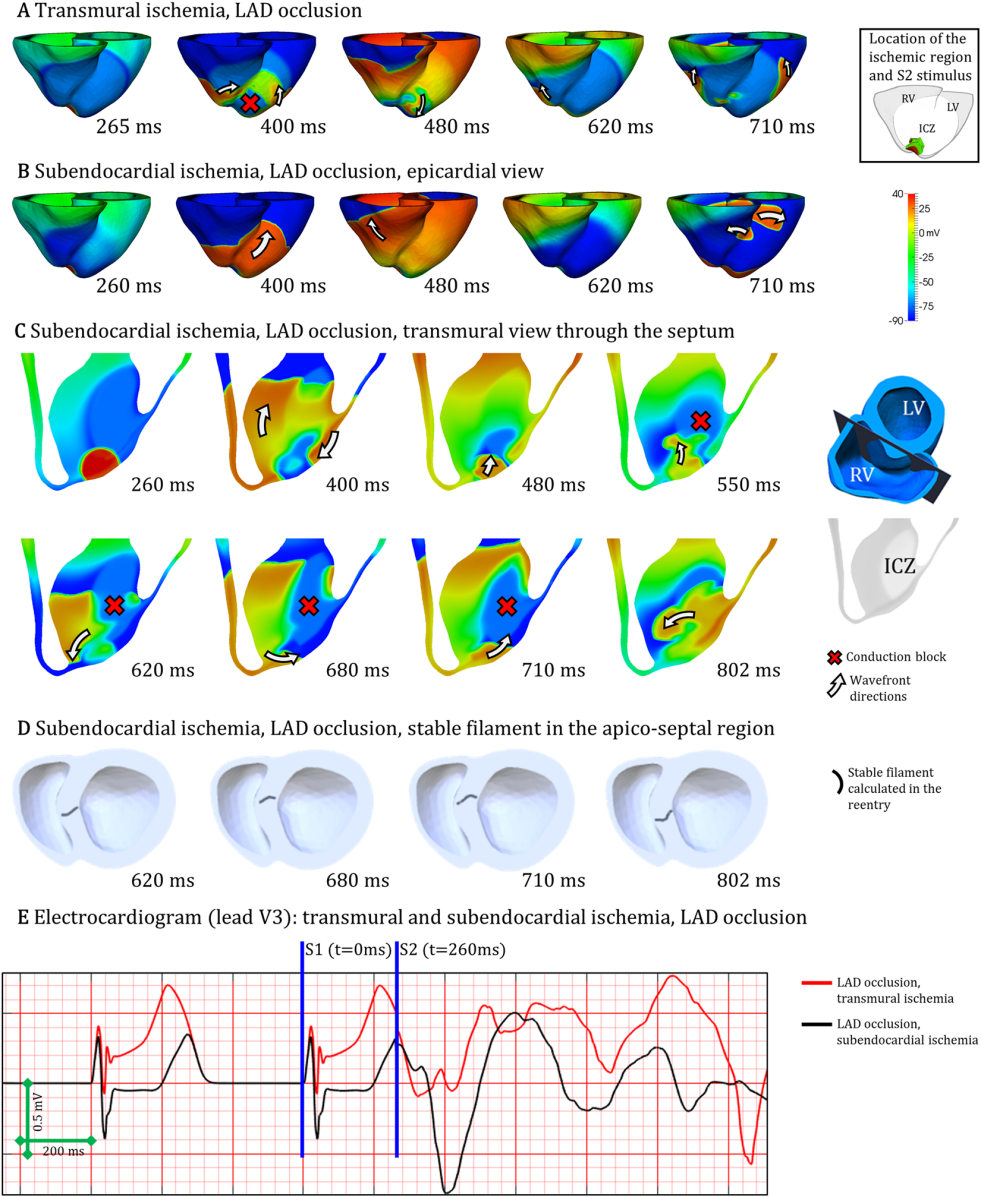
Two distinct mechanisms of reentry in acute regional ischemia. (**A**) Macro-reentry in transmural ischemia by LAD occlusion. (**B**) Transmural micro-reentry in subendocardial ischemia following LAD occlusion, epicardial view. (**C**) Same case as B, transmural view through the septum displaying the reentrant circuit. (**D**) Same case as B and C, visualization of the stable I-type filament located in the septo-apical region. (**E**) Electrocardiogram computed for both cases A and B/C/D. CI = 265 ms in A, CI = 260 ms in B/C/D.

As reported by Janse *et al*.^5^, an additional mechanism consisting of transmural micro-reentry was identified (Fig. 6, dark grey boxes), for both LAD and LCX occlusion. Even though transmural micro-reentries occurred both in transmural and subendocardial ischemia, they were the key mechanism explaining vulnerability to reentry in subendocardial ischemia (Fig. 6). Figure 7 demonstrates the differences in reentrant patterns in transmural versus subendocardial ischemia. As in Figs 5A,C, 7A illustrates the different stages of macro-reentry, with unidirectional conduction block in the ICZ, followed by propagation around the BZ (400 ms) proceeding retrogradely into the ICZ (480 ms) and finally reentering into the NZ (620 ms). The reentrant wave continues (710 ms), sustaining the macro-reentry around the ICZ.

However, in subendocardial ischemia (Fig. 7B), the epicardial view shows that the ectopic stimulus propagates through the ventricles (260 to 480 ms), with subsequent recovery (620 ms) until a breakthrough occurs (710 ms). In contrast, intramural dynamics are very different as shown in the transmural view through the septum in Fig. 7C. Following the ectopic stimulus (260 ms), propagation is blocked unidirectionally (red cross) in the subendocardial ischemic region (400 ms) but proceeds towards the base and also surrounds the ischemic region through the LV wall towards the apex (white arrows). Propagation continues to surround the BZ (480 ms, 550 ms), blocked in the ischemic region due to refractoriness (red cross), but reentering through normal tissue towards the apex (white arrows, 620 ms). It is then that a spiral wave is established, anchored in the BZ in the septo-apical region (Fig. 7C, 620 ms–802 ms). Figure 7D demonstrates the existence of a stable I-type filament in the septo-apical region. Simulated electrocardiographic signal in lead V3 of both reentries analysed in Fig. 7A,B/C/D are shown in 7E, associating macro reentries with larger wave amplitude than transmural micro-reentries (~1.3 versus ~0.5 mV).

**Figure 8:**
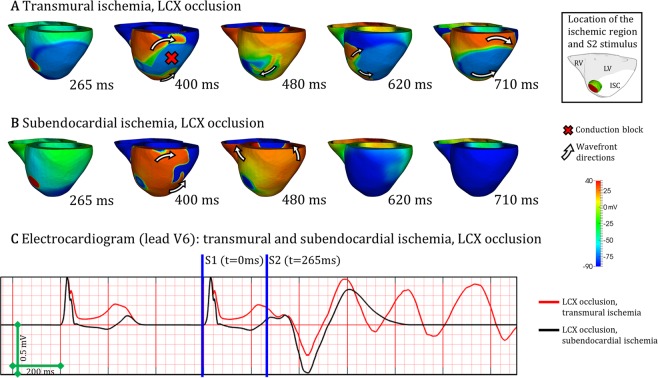
Reentrant mechanisms in LCX occlusion. (**A**) Macro-reentry in transmural ischemia by LCX occlusion. (**B**) No reentry in subendocardial ischemia by LCX occlusion. (**C**) Electrocardiogram computed for both cases A and B. CI = 265 ms in A and B.

The mechanisms underlying the smaller number of reentries in subendocardial versus transmural ischemia post-LCX occlusion are illustrated in Fig. 8A,B. Macro-reentry around the transmural ischemic region is established in Fig. 8A, similarly to the patterns shown in Fig. 7A. However, the subendocardial ischemic region is not able to sustain the conditions for reentry establishment, as shown in Fig. 8B. Figure 8C shows the electrocardiogram signal simulated in lead V6 for the cases displayed in Fig. 8A,B. More details about the formation of reentries can be found in the Supplementary Videos (SV1–SV4).

## Discussion

In this simulation study, the human ventricles affected by acute regional ischemia are shown to sustain two key types of reentrant patterns that determine differences in arrhythmic risk with varying ischemic region location, transmurality and size using high performance simulations. Macro-reentry around the ischemic region occurs primarily for large fully-transmural ischemic regions leading to ST elevation, for both LAD and LCX occlusion. Large ischemic regions and slow myocardial propagation favoured the establishment of macro-reentries. Furthermore, transmural micro-reentry at the ischemic border zone was identified as critical to explain arrhythmic risk, particularly for subendocardial ischemic regions, associated with mild ST depression.

Our simulation results also show a higher arrhythmic risk in LAD versus LCX post-occlusion, for the conditions tested, in agreement with clinical reports^34^. Simulations reveal that this is due to a higher propensity for the establishment of transmural reentrant circuits at the intersection of ischemic region and septo-apical region in LAD occlusion, rather than in the LV for LCX occlusion^8,11^. Furthermore, simulations show that QRS downslopes may provide useful information on ischemic extent for subendocardial ischemia, whereas ST elevation is an indicator of size for fully-transmural ischemic regions^19,35^.

The credibility of the findings is supported by the consistency of the simulation results with experimental and clinical recordings at the different scales involved, as proposed by Carusi *et al*.^36^. Firstly, ischemic tissue displays key electrophysiological alterations known to determine ECG biomarkers and reentrant patterns post-occlusion, and specifically prolonged post-repolarization refractoriness and slow conduction velocity^3,4,16,24,25^. This also includes electrophysiological gradients in the ischemic border zone, as in our previous studies^15,26,28^. Furthermore, the activation sequence used to simulate sinus rhythm has also been shown to be consistent with experimental recordings^29^. Finally, ECG patterns in control and in acute ischemia are in agreement with clinical 12-lead ECGs recordings^21,32,33^. This is both in terms of QRS complex progression between precordial leads (Supplementary Fig. SF2) and ischemia-induced alterations in QRS slopes and ST elevation (Fig. 2), shown to be in the range with values reported in clinical studies^35,37^.

The simulations with the human ventricular acutely-ischemic model also reproduce the figure-of-eight reentrant patterns around the ischemic region, as reported experimentally by^5,15^. These macro-reentries are favoured by large transmural ischemic regions, and their likelihood is explained by the wavelength for reentry. Simulations also reveal micro-reentries, also reported experimentally^5^, which are facilitated by slow intramural conduction close to the BZ. Whereas they also occur in transmural ischemia, micro-reentries are the only observed mechanism of reentry in subendocardial ischemia. Post-LAD occlusion, the intersection of the BZ and the septo-apical region favours the establishment of micro-reentry by providing larger intramural pathways than in the LV free wall. This can explain the higher arrhythmic risk in LAD versus LCX occlusion^8,11,38^. The existence of two pro-arrhythmic mechanisms of reentry in acute ischemia determined by the transmurality and location of the ischemic region may offer opportunities for targeted anti-arrhythmic treatments.

Slow propagation in the remote myocardium had a substantial effect on arrhythmic risk in our simulations, even though with negligible ECG effects (Fig. 4). Fibrosis^39^, diabetes^40^, or hypertrophy^41^ are main risk factors potentially affecting cell coupling and consequently decreasing myocardial conduction, albeit with unclear ECG signature^42^. In our simulations, slow conduction promoted reentry in acute regional ischemia. This is in close correspondence with *ex-vivo* studies linking the occurrence of transmural reentries to conduction delays produced by fibrotic tissue^43^. It could also explain that patients suffering from fibrosis as a consequence of pathologies like hypertrophic cardiomyopathy^44^ or diabetes mellitus^45^ are at higher risk under ischemic conditions.

Moreover, subendocardial ischemia was associated with slight ST depression in our simulations, as in Wilhelms *et al*.^46^, but also with high arrhythmia vulnerability, for LAD occlusion. The significance of potential risk predictors in silent myocardial ischemia remains an open discussion^47,48^. Significant ST elevation as main clinical marker of acute ischemia severity^48^ was only correlated in our results with fully-transmural scenarios supporting macro-reentry (Fig. 3). Conversely, changes in QRS downslope^19^ accurately represented the proportion of affected tissue and arrhythmic risk for LAD occlusion.

## Limitations of the Study

The consequences of acute myocardial ischemia are complex and dynamic. In this study we focus on simulating the electrophysiological consequences of the first 10 minutes post-occlusion on action potential, refractoriness and conduction velocity to investigate ECG alterations and reentry vulnerability in one human ventricular anatomical model of phase 1A ischemia. In addition to these factors, variability in anatomy, perfusion areas, ischemic region shape and severity, and baseline electrophysiological properties could also modulate arrhythmic risk^49–52^. Additional factors to consider are that simulations are undertaken in a stationary (non-contracting) ventricular model, and anatomical features such as endocardial trabeculations, papillary muscles and autonomic balance could play a role in reentrant pathways during ischemia^53–56^. Our study did not consider the role the Purkinje system may have during reentrant activity in acute ischemia. Building on the new insights provided by our study, further studies can investigate those additional factors.

## Conclusion

In this study, we present a human torso/biventricular modelling and simulation study into the mechanisms of variability in arrhythmic risk and ECG biomarkers in acute regional ischemia (phase 1A). The credibility of the simulation findings is supported by the consistency of simulated electrophysiological properties obtained with the human multiscale model with experimental and clinical recordings for ionic, cellular, tissue, and ECG properties. We identified transmural micro-reentries, in addition to the established macro-reentry around the ischemic region, as key pro-arrhythmic mechanisms in acute regional ischemia. Transmural acute ischemia (causing ST elevation) led to both macro- and micro-reentries, whereas arrhythmic risk in subendocardial ischemia (associated with mild ST depression) was predominantly explained by transmural micro-reentry. The modelling and simulation technology and results presented can inform the safety and efficacy evaluation of anti-arrhythmic therapy in acute myocardial ischemia.

## Supplementary information

Supplementary materials

Supplementary video SV1

Supplementary video SV2

Supplementary video SV3

Supplementary video SV4
