# Supplementary material for: High arrhythmic risk in antero-septal acute myocardial ischemia is explained by increased transmural reentry occurrence
Source: Sci Rep. 2019 Nov 14;9:16803. doi: 10.1038/s41598-019-53221-2 (PMC6856379; doi:10.1038/s41598-019-53221-2)
Supplement: Supplementary file 1 — Supplementary materials [file 41598_2019_53221_MOESM1_ESM.docx]

## High arrhythmic risk in antero-septal acute myocardial ischemia is explained by increased transmural reentry occurrence

## Supplementary material

## Authors:

Hector Martinez-Navarro^a^, MSc (hector.martinez-navarro@cs.ox.ac.uk),

Ana Mincholé^a^, PhD (ana.minchole@cs.ox.ac.uk),

Alfonso Bueno-Orovio^a^, PhD (alfonso.bueno@cs.ox.ac.uk),

*Blanca Rodriguez^a^, PhD (blanca.rodriguez@cs.ox.ac.uk)

^a^Department of Computer Science, British Heart Foundation Centre of Research Excellence, University of Oxford, Parks Rd., OX13QD, Oxford, UK

### Expanded methods

**EM.1. Human torso/biventricular model in regional acute myocardial ischemia**

An anatomically-based multiscale human torso/biventricular model in acute regional ischemia was constructed and evaluated based on extensive experimental and clinical data.^1–4^ To simulate sinus rhythm, a realistic activation sequence^5^ was implemented as described in ^6^, based on a fast conduction system mimicking propagation through the subendocardial Purkinje network at a propagation speed of 140 cm/s. This yielded realistic QRS complexes in the 12 lead ECG. Three sinus rhythm stimuli were applied, and the second and third beats produced consistent ECG biomarkers. The same endocardial activation sequence was applied for control and ischemic conditions, given endocardial tissue receives oxygen and nutrients through the blood in the ventricular cavity.^4^ An extra-stimulus S2 was applied at varying coupling interval (CI)^7–9^ between 225 and 300ms to identify reentry occurrence. This was primarily due to the fact that our study concerns investigating the mechanisms of establishment of re-entry, rather than the transition from re-entry to fibrillation. The S2 stimuli were applied to the full transmural extent of the myocardial wall consistent with the experimental observations in ^10^. Thus, S2 stimuli was modelled as a local ectopy propagating across the myocardial tissue, not originated from or propagated through the fast endocardial layer.

Following the S2 application, Purkinje cardiomyocytes are likely to be refractory given the fact that action potential duration and refractory period is longer in Purkinje than in ventricular cardiomyocytes.^11^ Therefore, the participation of the Purkinje system in the establishment of the re-entry is unlikely. This is further supported by the fact that the patterns of re-entry observed, especially with respect to macro-reentry, are in agreement with those reported experimentally. Further experimental and simulation studies are needed to model and simulate the role of Purkinje in arrhythmogenesis in acute ischemia.

Fibre orientation was imposed in the myocardial mesh with a rule-based method reproducing the experimental findings by Streeter et al.^12^ Biophysically-detailed human membrane kinetics were simulated using the modified version of the O’Hara-Rudy (ORd) model^13^ for ischemic conditions proposed by Dutta et al.^14^ The modified model includes the formulation of the ATP-dependant potassium current I_K(ATP)_^15^, and a modified sodium channel formulation based on previous studies^14,16^ to overcome the limitations of the original model in reproducing post-repolarization refractoriness in ischemic cells. The modified version of the ORd model includes a total of 41 ordinary differential equations (ODEs) describing ion channel dynamics and intracellular calcium handling in human ventricular electrophysiology.

The bidomain equations were used to describe electrical conduction in the ventricular and torso domains, given by

$$\begin{matrix} \nabla\cdot\left( \sigma_{i}\nabla\phi_{i} \right)=\beta\left( C_{m}\frac{\partial V}{\partial t}+I_{ion} \right)-I_{stim}, & \mathrm{in}\Omega_{h}, \\ \nabla\cdot\left( \sigma_{e}\nabla\phi_{e} \right)=-\beta\left( C_{m}\frac{\partial V}{\partial t}+I_{ion} \right), & \mathrm{in}\Omega_{h}, \\ \nabla\cdot\left( \sigma_{t}\nabla\phi_{e} \right)=0, & \mathrm{in}\Omega_{t}, \end{matrix}$$

where $\Omega_{h}$ and $\Omega_{t}$ are the heart and torso domains; $\phi_{i}$ and $\phi_{e}$ (in mV) represent the intracellular and extracellular potentials, respectively;$\sigma_{i}$ and $\sigma_{e}$ (in mS·cm^-1^) the conductivity tensors associated to the ventricular intracellular and extracellular domains, and $\sigma_{t}$ (in mS·cm^-1^) the torso conductivity; $\beta$ (set to 1400 cm^-1^) is the cellular membrane surface to volume ratio; and $C_{m}$ (set to 1 µF·cm^-2^) is the cell membrane capacitance. In these equations, $V$ is the membrane transmembrane potential, given by the difference between $\phi_{i}$ and $\phi_{e}$. $I_{ion}$ (in Am^-2^) is the current resulting from ionic channels, pumps and exchangers, as obtained from the modified version of the ORd model^14^, and the stimulus current $I_{stim}$ is imposed at each point of the endocardial surface by the activation sequence^6^, with a cycle length of 600 ms. The bidomain system of partial differential equations (PDEs) is closed with appropriate non-flux Neumann boundary conditions, representing conservation of current: $\vec{n}\cdot\left( \sigma_{i}\nabla\phi_{i} \right)=0$ on $\partial\Omega_{h}$, and $\vec{n}\cdot\left( \sigma_{t}\nabla\phi_{e} \right)=0$ on $\partial\Omega_{t}\backslash\partial\Omega_{h}$, where $\partial\Omega_{h}$ is the ventricular surface, $\partial\Omega_{t}\backslash\partial\Omega_{h}$ is the external torso surface, and $\vec{n}$ is the exterior normal vector to the domains.

In this work, the ratio of longitudinal to transverse values of the components of the myocardial intracellular and extracellular conductivity tensors was based on experimental data.^17^ To account for realistic conduction velocity in human using the ORd model, conductivity tensors were optimised by upscaling previous values^6^ by a factor of 1.8, yielding a longitudinal conduction velocity of 65 cm/s, in agreement with experimental data in human myocardium as reported by Taggart et al.^3^ This resulted in conductivity tensors $\sigma_{i}=\left[ 2.7, 0.81, 0.45 \right]$ and $\sigma_{e}=[9.828, 3.654, 3.654]$ (mS·cm^-1^), representing the conductivities in the longitudinal, transverse and normal directions, respectively. By upscaling the mentioned values^6^ by a factor of 1.2, instead of 1.8, we modelled slow myocardial propagation, reducing conduction velocities approximately 25% (Table ST1). In addition to these, the torso mesh includes defined volumes for lung and bone regions, with specific isotropic conductivities for simulating different materials. Following Cardone-Noott et al.^6^, the following values were used: $\sigma_{torso}=2.16$, $\sigma_{bone}=0.2$, $\sigma_{lung}=0.389$ (mS·cm^-1^).

The bidomain equations were discretized in space using a volumetric tetrahedral mesh, and solved with the Finite Elements Method (FEM) in Chaste.^18^ The spatial discretization of the myocardium was 0.4 mm between nodes in average, which ensured numerical convergence^6,19^, resulting in a total of 2.51 million nodes and 14.2 million tetrahedral elements. The combined heart-torso mesh consists of about 3.25 million nodes and 19.4 million tetrahedra. The ODE system representing cellular electrophysiology was solved in each spatial point using CVODE, a software package for stiff ODEs and variable-order and variable-step multistep methods, and a maximum ODE timestep of 0.02 ms. The PDE system was advanced in time using the same timestep of 0.02 ms and an implicit Euler stencil, as described.^18^

Transmural heterogeneities were implemented in the myocardium to yield realistic T wave morphologies in the computed 12-lead ECG from the simulations. A parameter sensibility analysis was performed and the epicardial, mid-myocardial and endocardial layers were set to 30%, 25% and 45% of the myocardial width, respectively. Given their importance in the formation of T waves in the ECG^20^, the myocardial model further includes apico-basal heterogeneities based on changes in the delayed rectifier (I_Ks_) current.^21^ By establishing an exponential gradient scaling original I_Ks_ conductance by 5.0 in the apex to 0.2 in the base, action potential duration is 40 ms shorter in the apical than in the basal region.^22^ Virtual electrodes were positioned on the torso at standard electrode locations for the calculation of the 12-lead ECG.^6^

In the simulations of re-entry, we computed the corresponding filaments (i.e. centres of organisation of the re-entry), as described by Fenton and Karma.^23^ Filament lines were calculated as the intersection line of two surfaces: an isopotential surface of constant membrane potential (-20 mV) and the isosurface established by membrane potential gradient is zero:

$$V-V_{iso}=\partial_{t}V= 0$$

### EM.2 Electrophysiological heterogeneities in the ischemic region

In our study, we investigated the implications of variability in size, location and transmurality of the ischemic region. Variability in location was investigated by considering ischemic regions in the anterior and lateral myocardial walls, representing LAD and LCX occlusion, respectively. Subendocardial ischemia was set to affect the subendocardial half of the myocardium, whereas transmural ischemia affected its full extent (Figure SF1, panel A). Two sizes were defined of 6 and 3 cm diameters, respectively, as in Dutta et al.^19^, by intersecting a sphere with the myocardial volume, in order to provide similar extents for all locations and transmurality. The resulting extent of the ischemic regions is in line with the relevant clinical ranges reported in the literature.^24,25^ For post LCX occlusion, the ischemic region covers from 2.8% (for subendocardial, 3cm diameter region) to 40.3% (for transmural, 6cm diameter region) of the LV volume, in agreement with 13-72% reported in ^24^. In terms of whole-ventricular volume, ischemic areas considered cover 1.2 to 17% for LCX occlusion, and 2.4 to 20% for LAD occlusion, which are clinically relevant with respect to the threshold of >10% recommended for revascularization and is in line with clinical data.^26^

Electrophysiological alterations in the ischemic region were modelled including: i) the ischemic core zone (ICZ); ii) the lateral border zone (BZ); and iii) the endocardial BZ, as described experimentally^4,10,27^ (Figure SF1, panel B). In the ICZ, ionic changes resulting from the main electrophysiological effects of acute ischemia (hyperkalemia, hypoxia and acidosis) were introduced. We applied a degree of ischemic severity that corresponds to highest arrhythmic risk^28^, and which coincides with early stages of ischemia (up to 15 min). Model values are based on the following experimental findings:^4,27,29,30^ Ischemic cells suffer from increased extracellular potassium concentration ([K^+^]_o_=9.5 mmol/L), increased I_K(ATP)_ by 7% (caused by hypoxia), and decreased peak conductance of fast sodium (I_Na_) and L-type calcium currents (I_CaL_) by 25% (due to acidosis). The BZ was modelled as in previous computational studies^19,31,32^, providing a linear transition in ischemic parameters between the ICZ and the normal zone (NZ) tissue, as shown experimentally.^4,10^ NZ tissue had a [K^+^]_o_ of 5.4 mmol/L (baseline value in the ORd model), zero I_K(ATP)_ and default conductances for I_Na_ and I_CaL_ as in the ORd model. Ischemia-induced action potential duration shortening, resting potential elevation and ERP prolongation reported in Table ST2 are in agreement with experimental recordings in acute ischemic conditions^2,3^, as also analysed in ^14^. Simulations using the human model also replicate the increase in post-repolarization refractoriness reported in acute myocardial ischemia^2,9,14,33,34^, with values in the experimental range of 60-150ms reported by ^2^ and further investigated by ^14^.

**EM.3 Comparison of simulated and clinical ECG biomarkers**

As explained in the first section of the Results section, the human torso-biventricular model used in this study was constructed and evaluated with a wide range of experimental recordings.^3–5,10,27^ The human ventricular membrane kinetics in acute ischemia were investigated in our previous study at cellular and tissue levels.^14^ Our previous computational studies also demonstrated modelling and simulation of the heterogeneity across the ischemic border zone.^19,31,35^ Furthermore, in this study, we demonstrate the agreement of simulated ECG with clinical ECGs recordings both under healthy^36^ and importantly under subendocardial and transmural ischemic conditions. We consider ST deviation^37,38^ and variations in QRS downslopes^39,40^ as main clinical markers of acute ischemia severity. Figure SF2 represents the simulated ECG under healthy conditions compared with a representative healthy volunteer ECG recording (<https://physionet.org/>^41^, PTB database, subject 173). The adjacent table contains the main ECG biomarkers computed under control healthy conditions. Clinical ECG recordings had investigational review board approval.

ST deviation was computed as the voltage deviation from baseline 60 ms after the J point as in other studies.^42,43^ The J point was determined manually as the end of the QRS complex, where the high-frequency components of the QRS complex transition to the low-frequency ST segment. QRS width was measured on leads V1 and V2 as in other studies.^44^ The resulting QRS width (80 ms) falls within the clinical range of QRS duration for healthy male and female populations as reported by Rijnbeek et al.^45^ Simulated electrophysiological dynamics are in agreement with reported activation sequences in human using microelectrode recordings, electromechanic wave imaging and electrocardiographic imaging.^6^

For QT calculations, the end of the T wave was defined as the intersection of the descending slope of its terminal part with the isoelectric potential. The resulting QT intervals were then corrected for heart rate using the Bazett’s formula (QTc = 420 ms), showing agreement with the clinical ranges observed in healthy populations.^45^ ST segment values from our computational model and the comparison against experimental data of humans under ischemic conditions are described in Results.

QRS_DS_ was computed as by Pueyo et al.^39^: after the point with the maximum slope is determined, a line is fitted in the ECG signal in windows of 15 ms centered around the point. Special consideration was taken to exclude any notch in the R wave from the 15 ms window used for the fitted line. Subsequently, certain cases were computed with smaller windows. The slope of the obtained line would be the maximum QRS_DS_. We define change in the maximum QRS downslope (ΔQRS_DS_) as the difference between the maximum QRS_DS_ value measured under ischemic conditions and the maximum value obtained in control conditions. We established that positive values in ΔQRS_DS_ indicate flatter QRS downslopes in comparison to the control scenario.

**Supplementary Tables**

|  | Longitudinal | Transversal | Propagation |
| --- | --- | --- | --- |
| Healthy | 64.5 | 30.5 | Normal |
|  | 47.7 | 22.7 | Slow |
| Ischemia | 40 | 20.5 | Normal |
|  | 32.4 | 15.8 | Slow |

**Table ST1.** Conduction velocities measured in simulations of electrical propagation in myocardial tissue fibres along the longitudinal and transversal axis, under ischemic and healthy conditions and with normal and slow myocardial propagation. Units expressed in cm/s.

| Cell model | APD90 | Resting potential | ERP |
| --- | --- | --- | --- |
| ORd modified as in Dutta et al. 2017, CL=600ms |  |  |  |
| Healthy | 240 ms | -88 mV | 231 ms |
|  |  |  |  |
| 10 min post occlusion | 169 ms | -73 mV | 252 ms |
| ([K^+^]_o_=9.5mM, f_K(ATP)_=0.07, I_Na_ and I_CaL_ conductances 25% reduced) |  |  |  |

**Table ST2.** Changes in action potential duration, resting potential and effective refractory period after 10 min of acute myocardial ischemia using the modified ORd model version by Dutta et al.^14^ Note the APD shortening, elevation of resting potential and ERP prolongation resulting in post-repolarization refractoriness, as reported in acute myocardial ischemia.^2,9,14^

**Supplementary Figures**

**
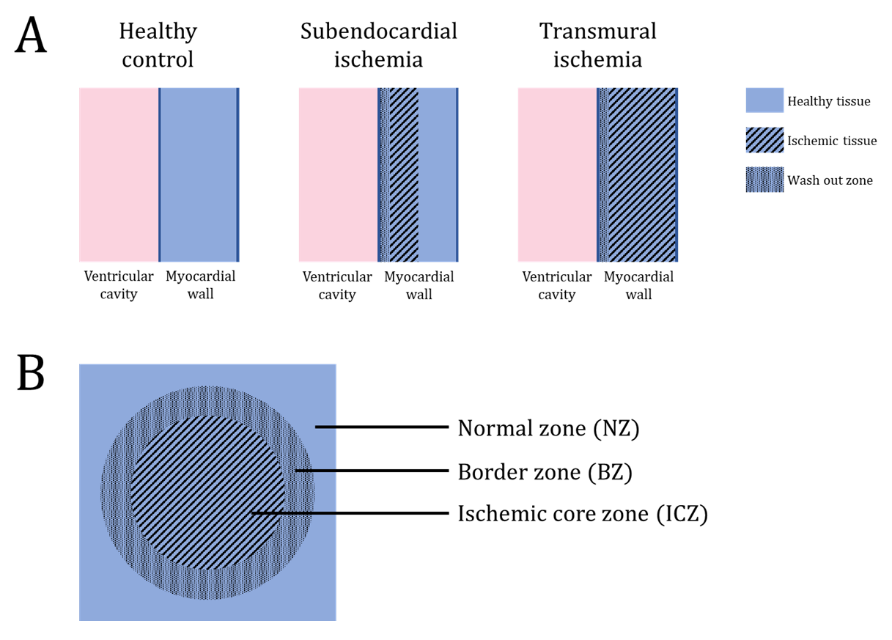
**

**Figure SF1.** Modelling of ischemic regions. A: Spatial distribution of ischemia through the myocardial wall for subendocardial and transmural ischemia. B: Schematic representation of heterogeneity in acute regional ischemia including normal, border and ischemic core zones.


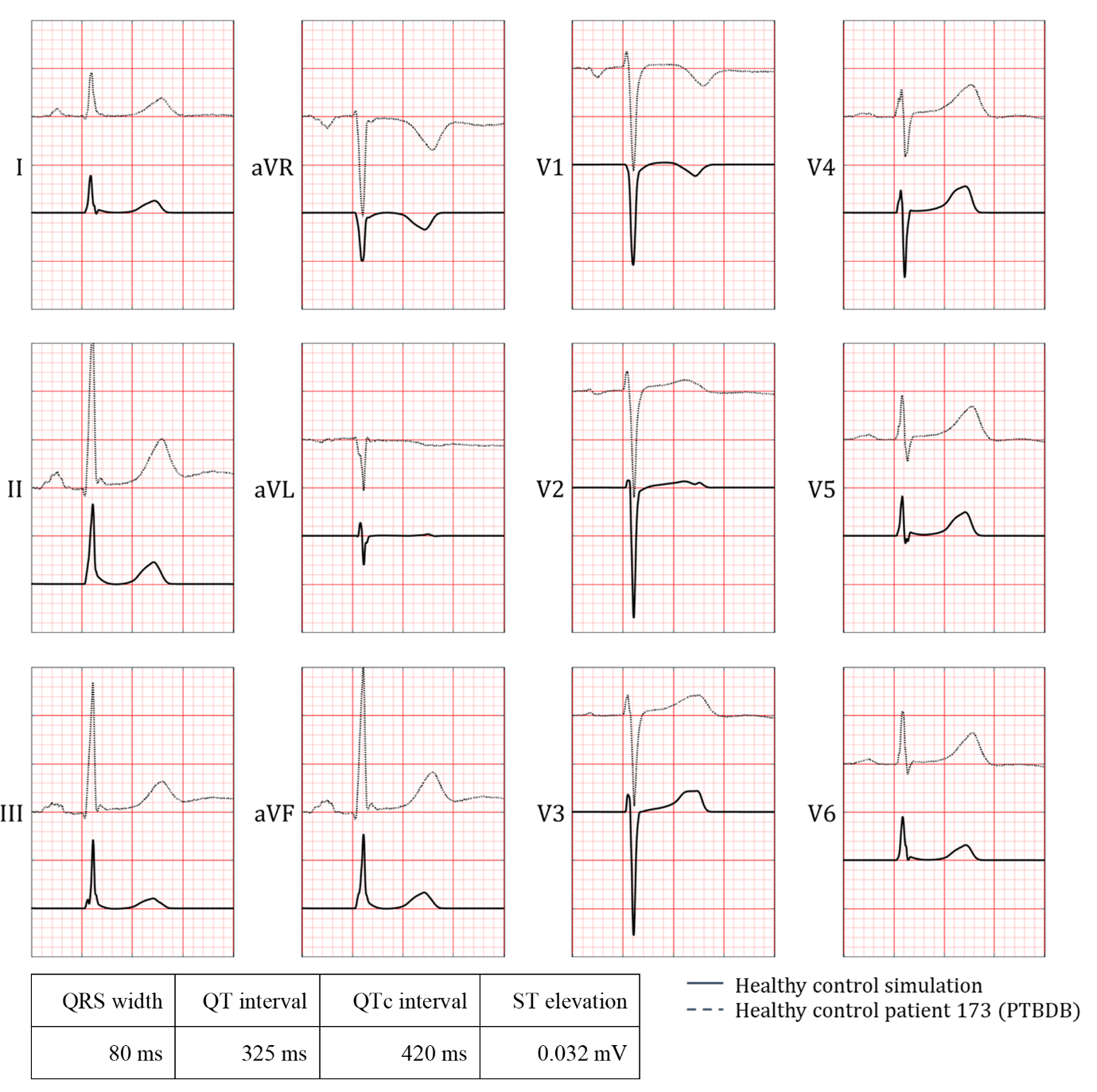


**Figure SF2.** Comparison of simulated ECG (black solid line) under control healthy conditions, with the 12-lead ECG recording from subject 173 in the PTB database (light grey line). Table displays the main ECG biomarkers measured in the simulation under healthy conditions and normal myocardial propagation.

<SV1.avi>

**Supplementary Video 1.** Macro-reentry in the human biventricular mesh following an ectopic beat in the septo-apical region of the border zone. Coupling interval: 265 ms; ischemic case: transmural ischemia under LAD occlusion (main text Figure 7A).

<SV2.avi>

**Supplementary Video 2.** Transmural micro-reentry in the human biventricular mesh following an ectopic beat in the septo-apical region of the border zone. Coupling interval: 260 ms; ischemic case: subendocardial ischemia under LAD occlusion (main text Figure 7B).

<SV3.avi>

**Supplementary Video 3.** Macro-reentry in the human biventricular mesh following an ectopic beat in the left-ventricular mid-cavity region of the border zone. Coupling interval; 265 ms; ischemic case: transmural ischemia under LCX occlusion (main text Figure 8A).

<SV4.avi>

**Supplementary Video 4.** Non-arrhythmogenic depolarisation sequence in the human biventricular mesh following an ectopic beat in the left-ventricular mid-cavity region of the border zone. Coupling interval: 265 ms; ischemic case: subendocardial ischemia under LCX occlusion (main text Figure 8B).

**References**

1. Carmeliet, E. Cardiac ionic currents and acute ischemia: from channels to arrhythmias. *Physiol. Rev.* **79**, 917–1017 (1999).

2. Sutton, P. *et al.* Repolarisation and refractoriness during early ischaemia in humans. *Heart* **84**, 365–369 (2000).

3. Taggart, P. *et al.* Inhomogeneous transmural conduction during early ischaemia in patients with coronary artery disease. *J. Mol. Cell. Cardiol.* **32**, 621–630 (2000).

4. Wilensky, R. L. *et al.* The subendocardial border zone during acute ischemia of the rabbit heart: an electrophysiologic, metabolic, and morphologic correlative study. *Circulation* **74**, 1137–1146 (1986).

5. Durrer, D. *et al.* Total Excitation of the Isolated Human Heart. *Circulation* **41**, 899–912 (1970).

6. Cardone-Noott, L., Bueno-Orovio, A., Mincholé, A., Zemzemi, N. & Rodriguez, B. Human ventricular activation sequence and the simulation of the electrocardiographic QRS complex and its variability in healthy and intraventricular block conditions. *Europace* **18**, iv4–iv15 (2016).

7. de Vries, L. J. *et al.* Coupling interval variability of premature ventricular contractions in patients with different underlying pathology: an insight into the arrhythmia mechanism. *J Interv Card Electrophysiol* **51**, 25–33 (2018).

8. Arevalo, H. J. *et al.* Arrhythmia risk stratification of patients after myocardial infarction using personalized heart models. *Nature Communications* **7**, ncomms11437 (2016).

9. Rodríguez, B., Trayanova, N. & Noble, D. Modeling cardiac ischemia. *Ann. N. Y. Acad. Sci.* **1080**, 395–414 (2006).

10. Janse, M. J. *et al.* Flow of ‘injury’ current and patterns of excitation during early ventricular arrhythmias in acute regional myocardial ischemia in isolated porcine and canine hearts. Evidence for two different arrhythmogenic mechanisms. *Circ. Res.* **47**, 151–165 (1980).

11. Dobrzynski, H. *et al.* Structure, function and clinical relevance of the cardiac conduction system, including the atrioventricular ring and outflow tract tissues. *Pharmacol. Ther.* **139**, 260–288 (2013).

12. Streeter, D. D., Spotnitz, H. M., Patel, D. P., Ross, J. & Sonnenblick, E. H. Fiber Orientation in the Canine Left Ventricle during Diastole and Systole. *Circulation Research* **24**, 339–347 (1969).

13. O’Hara, T., Virág, L., Varró, A. & Rudy, Y. Simulation of the undiseased human cardiac ventricular action potential: model formulation and experimental validation. *PLoS Comput. Biol.* **7**, e1002061 (2011).

14. Dutta, S., Mincholé, A., Quinn, T. A. & Rodriguez, B. Electrophysiological properties of computational human ventricular cell action potential models under acute ischemic conditions. *Progress in Biophysics and Molecular Biology* **129**, 40–52 (2017).

15. Michailova, A., Saucerman, J., Belik, M. E. & McCulloch, A. D. Modeling Regulation of Cardiac KATP and L-type Ca2+ Currents by ATP, ADP, and Mg2+. *Biophys J* **88**, 2234–2249 (2005).

16. Passini, E. *et al.* Mechanisms of pro-arrhythmic abnormalities in ventricular repolarisation and anti-arrhythmic therapies in human hypertrophic cardiomyopathy. *J Mol Cell Cardiol* **96**, 72–81 (2016).

17. Clerc, L. Directional differences of impulse spread in trabecular muscle from mammalian heart. *J. Physiol. (Lond.)* **255**, 335–346 (1976).

18. Pathmanathan, P. *et al.* A numerical guide to the solution of the bidomain equations of cardiac electrophysiology. *Progress in Biophysics and Molecular Biology* **102**, 136–155 (2010).

19. Dutta, S. *et al.* Early afterdepolarizations promote transmural reentry in ischemic human ventricles with reduced repolarization reserve. *Prog. Biophys. Mol. Biol.* **120**, 236–248 (2016).

20. Weiss, D. L. *et al.* Modeling of heterogeneous electrophysiology in the human heart with respect to ECG genesis. in *2007 Computers in Cardiology* 49–52 (2007). doi:10.1109/CIC.2007.4745418

21. Szentadrassy, N. *et al.* Apico-basal inhomogeneity in distribution of ion channels in canine and human ventricular myocardium. *Cardiovasc. Res.* **65**, 851–860 (2005).

22. Bishop, M. J., Vigmond, E. J. & Plank, G. The functional role of electrophysiological heterogeneity in the rabbit ventricle during rapid pacing and arrhythmias. *Am J Physiol Heart Circ Physiol* **304**, H1240–H1252 (2013).

23. Fenton, F. & Karma, A. Vortex dynamics in three-dimensional continuous myocardium with fiber rotation: Filament instability and fibrillation. *Chaos* **8**, 20–47 (1998).

24. Lee, J. T., Ideker, R. E. & Reimer, K. A. Myocardial infarct size and location in relation to the coronary vascular bed at risk in man. *Circulation* **64**, 526–534 (1981).

25. Edenbrandt, L. *et al.* Area of ischemia assessed by physicians and software packages from myocardial perfusion scintigrams. *BMC Med Imaging* **14**, 5 (2014).

26. Shaw Leslee J. *et al.* Optimal Medical Therapy With or Without Percutaneous Coronary Intervention to Reduce Ischemic Burden. *Circulation* **117**, 1283–1291 (2008).

27. Coronel, R., Wilms-Schopman, F. J., Opthof, T., van Capelle, F. J. & Janse, M. J. Injury current and gradients of diastolic stimulation threshold, TQ potential, and extracellular potassium concentration during acute regional ischemia in the isolated perfused pig heart. *Circ. Res.* **68**, 1241–1249 (1991).

28. Janse, M. J. & Wit, A. L. Electrophysiological mechanisms of ventricular arrhythmias resulting from myocardial ischemia and infarction. *Physiol. Rev.* **69**, 1049–1169 (1989).

29. Moréna, H. *et al.* Comparison of the effects of regional ischemia, hypoxia, hyperkalemia, and acidosis on intracellular and extracellular potentials and metabolism in the isolated porcine heart. *Circ. Res.* **46**, 634–646 (1980).

30. Vermeulen, J. T. *et al.* Electrophysiologic and extracellular ionic changes during acute ischemia in failing and normal rabbit myocardium. *J. Mol. Cell. Cardiol.* **28**, 123–131 (1996).

31. Tice, B. M., Rodríguez, B., Eason, J. & Trayanova, N. Mechanistic investigation into the arrhythmogenic role of transmural heterogeneities in regional ischaemia phase 1A. *Europace* **9 Suppl 6**, vi46-58 (2007).

32. Ferrero, J. M., Trénor, B., Rodríguez, B. & Sáiz, J. Electrical activity and reentry during acute regional myocardial ischemia: insights from simulations. *Int. J. Bifurcation Chaos* **13**, 3703–3715 (2003).

33. Downar, E., Janse, M. J. & Durrer, D. The effect of acute coronary artery occlusion on subepicardial transmembrane potentials in the intact porcine heart. *Circulation* **56**, 217–224 (1977).

34. Kodama, I., Wilde, A., Janse, M. J., Durrer, D. & Yamada, K. Combined effects of hypoxia, hyperkalemia and acidosis on membrane action potential and excitability of guinea-pig ventricular muscle. *J. Mol. Cell. Cardiol.* **16**, 247–259 (1984).

35. Trénor, B., Ferrero, J. M., Rodríguez, B. & Montilla, F. Effects of pinacidil on reentrant arrhythmias generated during acute regional ischemia: a simulation study. *Ann Biomed Eng* **33**, 897–906 (2005).

36. Bousseljot, R., Kreiseler, D. & Schnabel, A. Nutzung der EKG-Signaldatenbank CARDIODAT der PTB über das Internet. *Biomedizinische Technik/Biomedical Engineering* **40**, 317–318 (2009).

37. Jager, F. *et al.* Long-term ST database: A reference for the development and evaluation of automated ischaemia detectors and for the study of the dynamics of myocardial ischaemia. *Med. Biol. Eng. Comput.* **41**, 172–182 (2003).

38. Laguna, P. & Sörnmo, L. The STAFF III ECG database and its significance for methodological development and evaluation. *Journal of Electrocardiology* **47**, 408–417 (2014).

39. Pueyo, E., Sornmo, L. & Laguna, P. QRS slopes for detection and characterization of myocardial ischemia. *IEEE Trans Biomed Eng* **55**, 468–477 (2008).

40. Ringborn, M. *et al.* Evaluation of depolarization changes during acute myocardial ischemia by analysis of QRS slopes. *J Electrocardiol* **44**, 416–424 (2011).

41. Goldberger, A. L. *et al.* PhysioBank, PhysioToolkit, and PhysioNet. *Circulation* **101**, e215–e220 (2000).

42. Hasche, E. T., Fernandes, C., Freedman, S. B. & Jeremy, R. W. Relation Between Ischemia Time, Infarct Size, and Left Ventricular Function in Humans. *Circulation* **92**, 710–719 (1995).

43. Mather (Editor), P. J. *Jefferson Heart Institute Handbook Of Cardiology*. (Jones & Bartlett Publishers, 2010).

44. Blackburn, H. W. & Simonson, E. The total QRS duration. *American heart journal, American heart journal., The American Heart Journal, XIV International Congress on Glass - Collected Papers.* **53**, 699–710 (1957).

45. Rijnbeek, P. R. *et al.* Normal values of the electrocardiogram for ages 16-90 years. *J Electrocardiol* **47**, 914–921 (2014).
